# Clinicopathological Profile of Sinonasal Masses in a Tertiary Care Center in Central India: A Retrospective Study

**DOI:** 10.7759/cureus.50700

**Published:** 2023-12-17

**Authors:** Anjan K Sahoo, Shaila Sidam, Aparna Chavan

**Affiliations:** 1 Otolaryngology - Head and Neck Surgery, All India Institute of Medical Sciences, Bhopal, IND

**Keywords:** proptosis, inverted papilloma, angiofibroma, neoplastic, sinonasal

## Abstract

Introduction: Diseases of the nose and paranasal sinuses have a significant impact on the patient's functional and structural aspects. They affect all age groups and both sexes. Due to its proximity to vital structures, diseases of the nose and paranasal sinuses sometimes lead to very grave prognoses.

Materials and methods: This was a retrospective study done in one of central India's largest tertiary care centers. We studied 227 cases of sinonasal masses in both the non-neoplastic and neoplastic categories. Clinicopathological characteristics of masses were analyzed with the help of clinical and imaging findings and subsequently confirmed by tissue diagnosis.

Results: The mean age of presentation was 45.5 years, with a male-to-female ratio of 1.25:1. Most of our patients were from lower-middle socioeconomic groups with educational qualifications below the 10th standard. Nasal obstruction was the most common symptom. A computed tomography scan was the preferred radiological investigation. Sinonasal undifferentiated carcinoma was the most common variant of malignancy, with a total number of six out of 22 (27%).

Conclusion: The evaluation of sinonasal masses should be carried out systematically and meticulously. Since the initial presentation of most of the diseases of the nose and paranasal sinuses is the same, a proper clinical, radiological, and tissue diagnosis should be carried out to avoid causing malignant lesions.

## Introduction

Nasal and nasopharyngeal masses significantly impact the patient's functional and structural aspects. Diseases of the nose and paranasal sinuses are rising all over the world because of an increase in air pollution [1]. They affect all age groups and both sexes. Both benign and malignant conditions are commonly encountered during clinical practice. Because of its proximity to vital structures like the brain, orbit, and critical neurovascular structures, diseases of the nose and paranasal sinuses sometimes lead to very grave prognoses. Sinonasal masses like simple nasal polyps and their removal by Hippocrates were well mentioned in literature centuries ago [2].

All the sinonasal masses have typical signs and symptoms like nasal obstruction, nasal discharge, bleeding from the nose, facial swelling, decreased sense of smell, hearing loss, change in voice, etc. A detailed history of clinical examinations like nasal endoscopy, imaging, and histopathology collectively may lead to a conclusive diagnosis. Congenital midline nasal anomalies are rare and comprise gliomas, encephaloceles, dermoids, etc. Around 60% of nasal gliomas are extranasal, and 40% are intranasal [3]. Nasal polyposis, one of the most common inflammatory mass lesions of the nose, affecting up to 4% of the population, is smooth, semi-transparent, and pale in color and primarily arises from the mucosa of the osteomeatal complex [4]. Sinonasal malignancies comprise less than 5% of all head and neck malignancies, with somehow increasing trends presented with varied masked clinical presentation as that of benign disease poses a significant challenge to both patients and surgeons [5]. The purpose of this retrospective study in one of the largest tertiary care centers in central India is to provide a clinicopathological profile of various sinonasal masses.

## Materials and methods

This is a retrospective study of all the sinonasal masses presented to the Department of Otorhinolaryngology, All India Institute of Medical Sciences, Bhopal, India. This study included all patients diagnosed with sinonasal masses from July 2021 to August 2023. We retrieved all the data, such as age, sex, occupation, socioeconomic status, signs and symptoms, and endoscopic, imaging, and histopathological findings, from the medical record department. A total of 227 patients met our inclusion criteria. Patients with complete data on clinical and pathological findings were included. All the lesions were classified as non-neoplastic and neoplastic lesions, and again, the neoplastic lesions were divided into benign and malignant lesions. Institutional human ethics committee approval was obtained before commencing the study (approval number: IHEC-LOP/2023/IL0102). All the relevant history, including age, sex, personal, occupational, and socioeconomic history, and detailed clinical examinations, were retrieved. Patients were categorized as below 30 years, 30-50, 50-70, and above 70 years. Patients were also categorized as per their occupation, like farmers, businessmen, housemakers, students, etc. Details of symptoms and signs like nasal obstruction, nasal discharge, proptosis, periorbital swelling, vision loss, involvement of cranial nerves, bleeding from the nose, etc. were also collected and compiled. The anterior nasal endoscopy findings of all patients were compiled. The characteristics of mass, such as polypoidal, fleshy, or bleeding, during the procedures were also noted. The probing of all sinonasal masses and their findings were also collected. Significant findings like tumor site origin and tenderness on palpation were also reported. The imaging findings from both the CT scan and MRI were included in the study. The histopathology of 198 patients was retrieved and included in this study. All the patients were categorized as per the final histopathology into neoplastic and non-neoplastic categories. The neoplastic categories were again subdivided into benign and malignant categories. Three to four patients had immunohistochemistry for confirmation of the diagnosis. All the patients underwent surgery, radiotherapy, and medication per the standard treatment guidelines. Data was analyzed using Microsoft Office Excel 2010 (Microsoft Corporation, Washington, USA).

## Results

**Figure 1:**
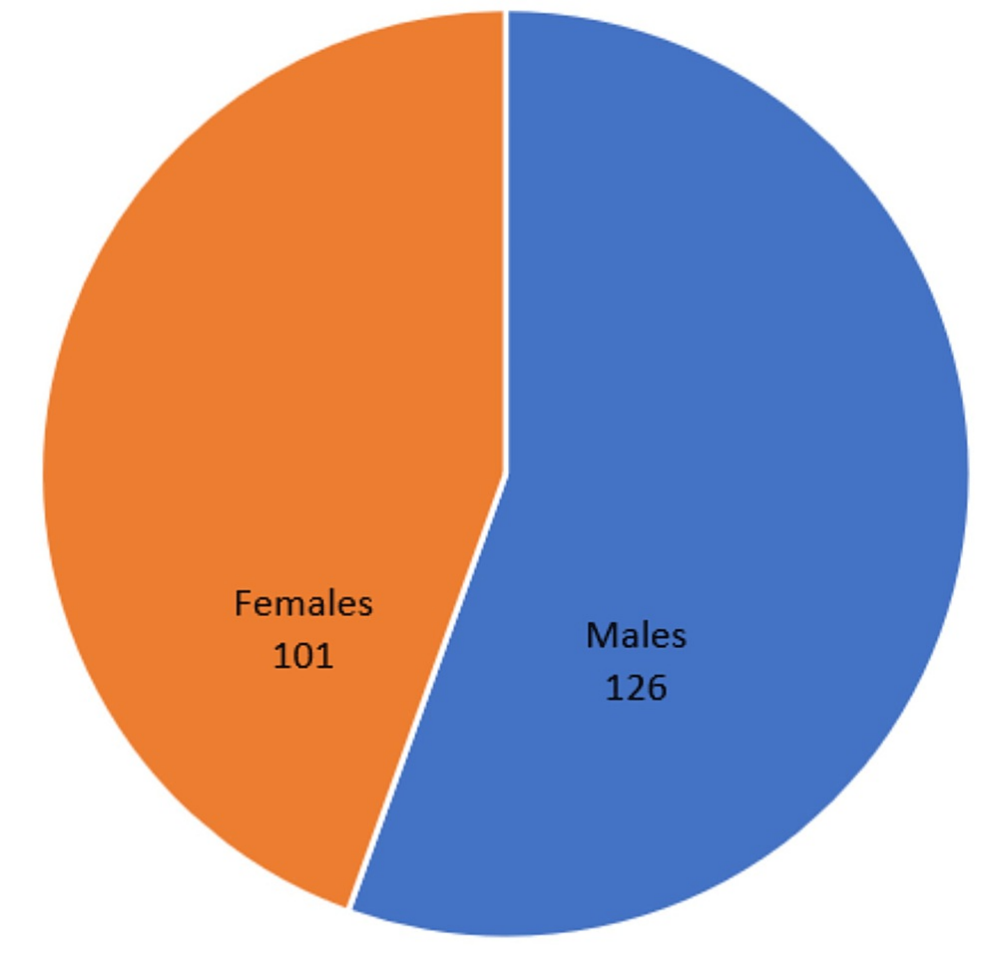
Sex ratio

**Figure 2:**
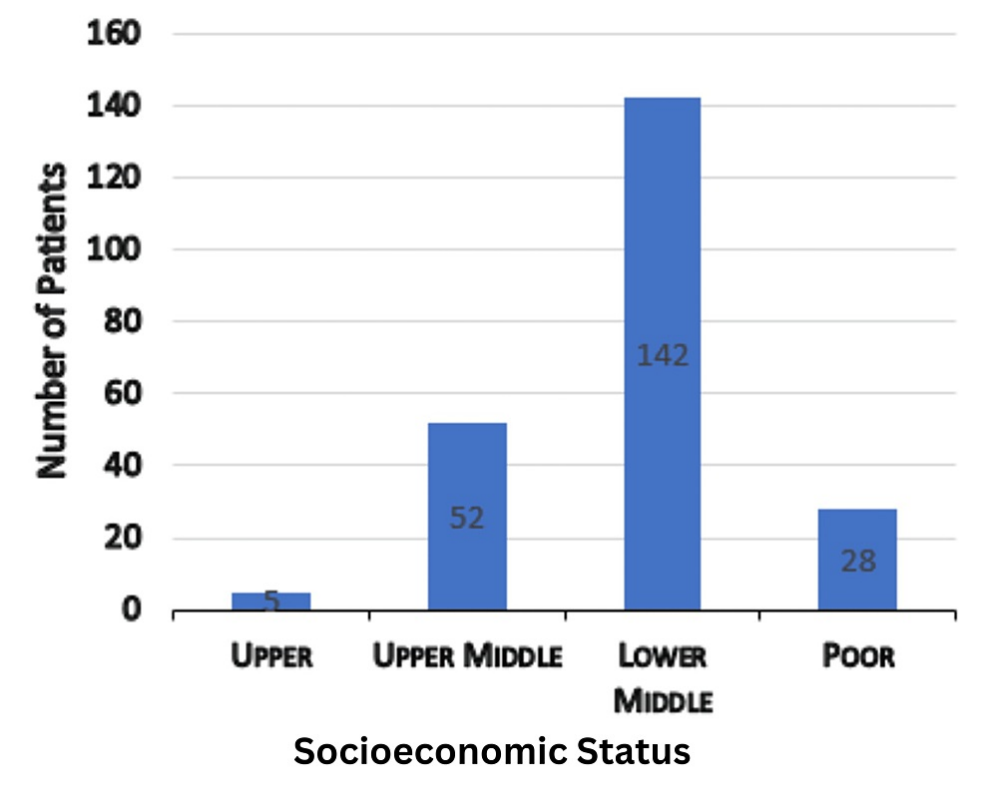
Socioeconomic status of patients

**Figure 3:**
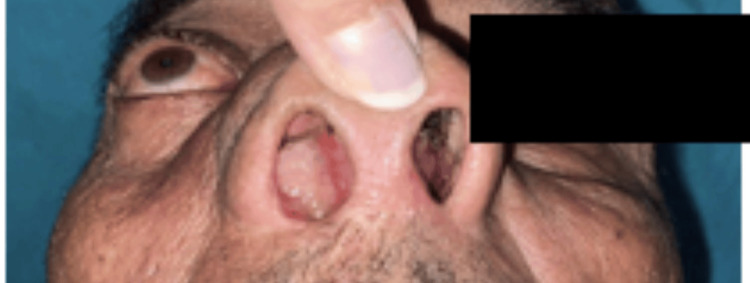
Clinical picture of unilateral nasal mass with proptosis

The age ranges from 3 to 82 years, with the maximum number of patients (32%, n=72) in the range of 50 to 70 years with a mean age of 45.5 years. There were 126 males and 101 females, with a male-to-female ratio of 1.25:1 (Figure 1). Most of our patients were housemakers (43%, n=98), followed by farmers (19%, n=44) (Table 1). Around 142 (63%) patients belong to the lower middle socioeconomic group, followed by 52 (23%) in the upper middle group (Figure 2). Nasal obstruction was the most common symptom (90%, n=204), followed by nasal discharge (74%, n=169). Abnormalities in extraocular movement and decreased vision were seen in 31 (14%) of cases, and total loss of vision in four (2%) of patients (Table 2). Proptosis was present in 15 (7%) patients (Figure 3). In 22 (10%) patients, a mass or bulging was present inside the oral cavity. On anterior rhinoscopy, 143 (63%) patients had septal deviations and spurs. The mass was polypoidal in 89 (39%) cases and fleshy in 31 (14%). Purulent secretions were present in 80 (35%) patients, and in 21 (9%) cases, there was bleeding during the process of anterior rhinoscopy. Probing of the nasal mass suggests that in 106 (47%) cases, the mass arose from the lateral nasal wall, and in 12 (5%) cases, it originated from the septum and floor. In 79 (35%) patients, there was bleeding on probing, and in 60 (26%) cases, it was tender to palpate. Almost all the patients except those with septal hematoma and inflammatory nasal polyps had a CT scan, MRI, or both as part of their radiological investigations. A CT scan was the preferred radiological investigation (85%, n=194). Noncontrast tomography was performed in 136 patients (60%), whereas contrast CT scans were done in 58 patients (25%). An MRI was performed on 69 patients (30%). Both CT and MRI were performed on 43 patients (19%). Histopathological examination was traced from 198 patients (87%) records. Of them, 139 patients were nonneoplastic, and 59 were neoplastic (22 were malignant, and 27 were benign) (Table 3). In the nonneoplastic category, 93 were fungal sinusitis, including mucormycosis, allergic fungal rhinosinusitis, and aspergillosis. Four patients (2%) had rhinosporidiosis. Diseases confined only to the frontal sinus were seen in six patients (3%). Out of 22 patients with sinonasal malignancy, six were of poorly differentiated carcinoma (27%), and four were of squamous cell carcinoma (18%). Sinonasal sarcoma was found in three patients (14%). Of the 27 benign neoplastic lesions, 10 were of juvenile nasopharyngeal angiofibroma (37%), and seven were of inverted papilloma (25%).

**Table 1:** Occupation of patients

Occupation	No. of patients (%)
Farmer	44 (19%)
Housemaker	98 (43%)
Service	28 (12%)
Business	16 (7%)
Students	29 (14%)
Others	12 (5%)

**Table 2:** Signs and symptoms

Symtoms	Number of patients (%)
Nasal obstruction	204 (90%)
Nasal discharge	169 (74%)
Sneezing	92 (40%)
Nasal bleed	63 (28%)
Facial swelling	54 (24%)
Facial pain	44 (19%)
Protrusion of eyeball	15 (7%)
Decrease sense of smell	28 (12%)
Change in voice	45 (19%)
Decrease vision	31 (14%)
Absent vision	4 (2%)
Hearing loss	6 (3%)
Restricted eye movement	31 (14%)
Mass/swelling in the oral cavity	22 (10%)

**Table 3:** Sinonasal masses included in the study CRS: chronic rhino sinusitis, AC: antrochoanal

Diagnosis	No. of patients (%)	Total
CRS with sinonasal polyposis	33 (14%)	227 (100%)
AC polyp	27 (12%)	
Fungal sinusitis	93 (40%)	
Rhinosporiodosis	4 (2%)	
Juvenile nasopharyngeal angiofibroma	10 (4%)	
Inverted papilloma	7 (3%)	
Nasolabial cyst	5 (2%)	
Rhinolith	1 (1%)	
Dentigerous cyst	2 (1%)	
Inflammatory polyp	3 (1%)	
Fibrous dysplasia	2 (1%)	
Frontal sinus disease	6 (3%)	
Nasal schwannoma	2 (1%)	
Hemangioma/hemangiopericytoma	2 (1%)	
Meningoencephalocele	1 (1%)	
Sphenochoanal polyp	1 (1%)	
Sinonasal malignancy	22 (10%)	
Septal hematoma/abscess	6 (3%)	

## Discussion

In our study, most sinonasal masses were in the age group of 50 to 70 years, whereas in other studies, the typical age group for sinonasal masses was in the range of the second to fifth decades [6]. Our study had a maximum elderly population, which may be because of the more significant number of invasive fungal sinusitis and malignancy patients in our study. Another study suggests that the peak age for benign, intermediate, and malignant lesions was in the second, fifth, and sixth decades, which also fits our study [7,8]. Contrary to the above, one study in Nigeria [2] suggested that the peak age for sinonasal malignancy is around 33 years. Sinonasal masses had a predilection for males, demonstrating a male (n=126) to female (n=101) ratio of 1.25:1. In the Indian study by Zafar et al. [9], it was greater (male-to-female ratio: 1.7:1), while in the Nigerian study [2], the ratio showed a preponderance of women (male-to-female ratio: 1:1.2). According to a British review [10], the male-to-female ratio for nasal polyposis was 2:1.

**Figure 4:**
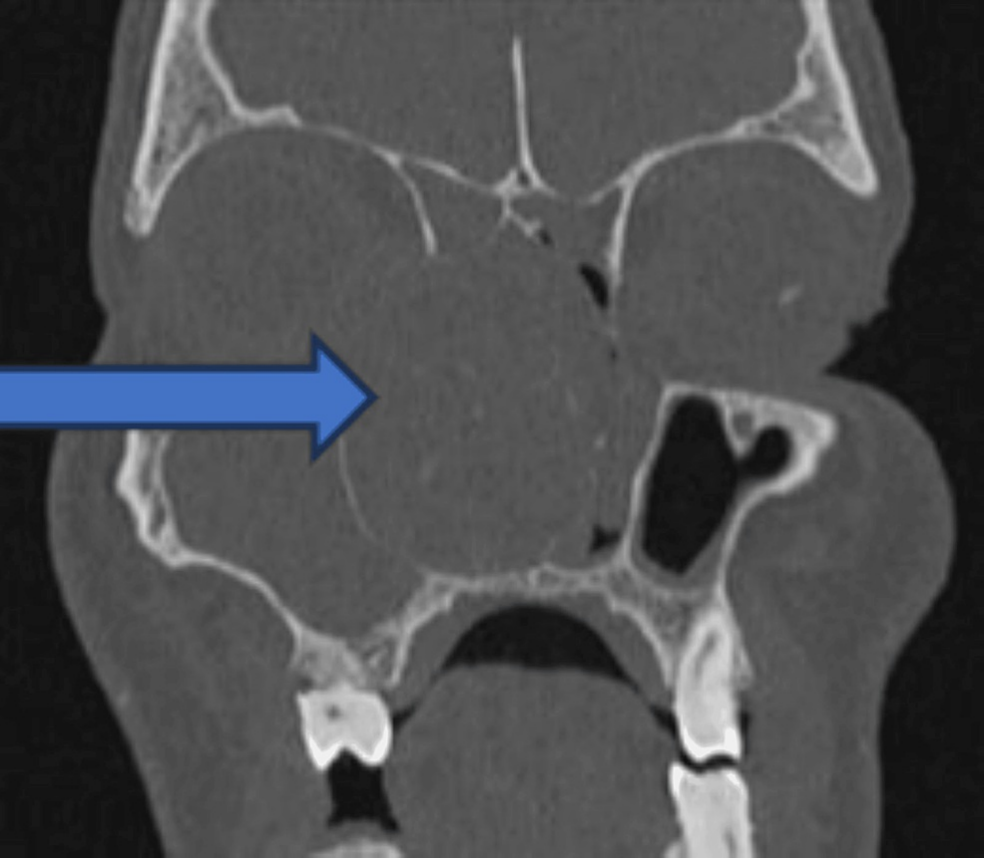
CT scan displaying a sizable, well-defined, smooth-walled lobulated soft tissue lesion centered in the right nasal cavity (solid arrow), measuring 5.6 x 4 x 4.8 cm, resulting in its expansion and protruding into the remaining right maxillary sinus. Erosion of the nasal septum with extension into the left nasal cavity is observed, reaching up to the osteomeatal complex. Superior extension encompasses both the anterior and posterior ethmoid sinuses and the floor of the bilateral olfactory fossa, with the cribriform plate not visualized. Moreover, a superolateral extension infiltrates the medial extraconal compartment of the right orbit, abutting the right medial rectus and superior oblique muscles

**Figure 5:**
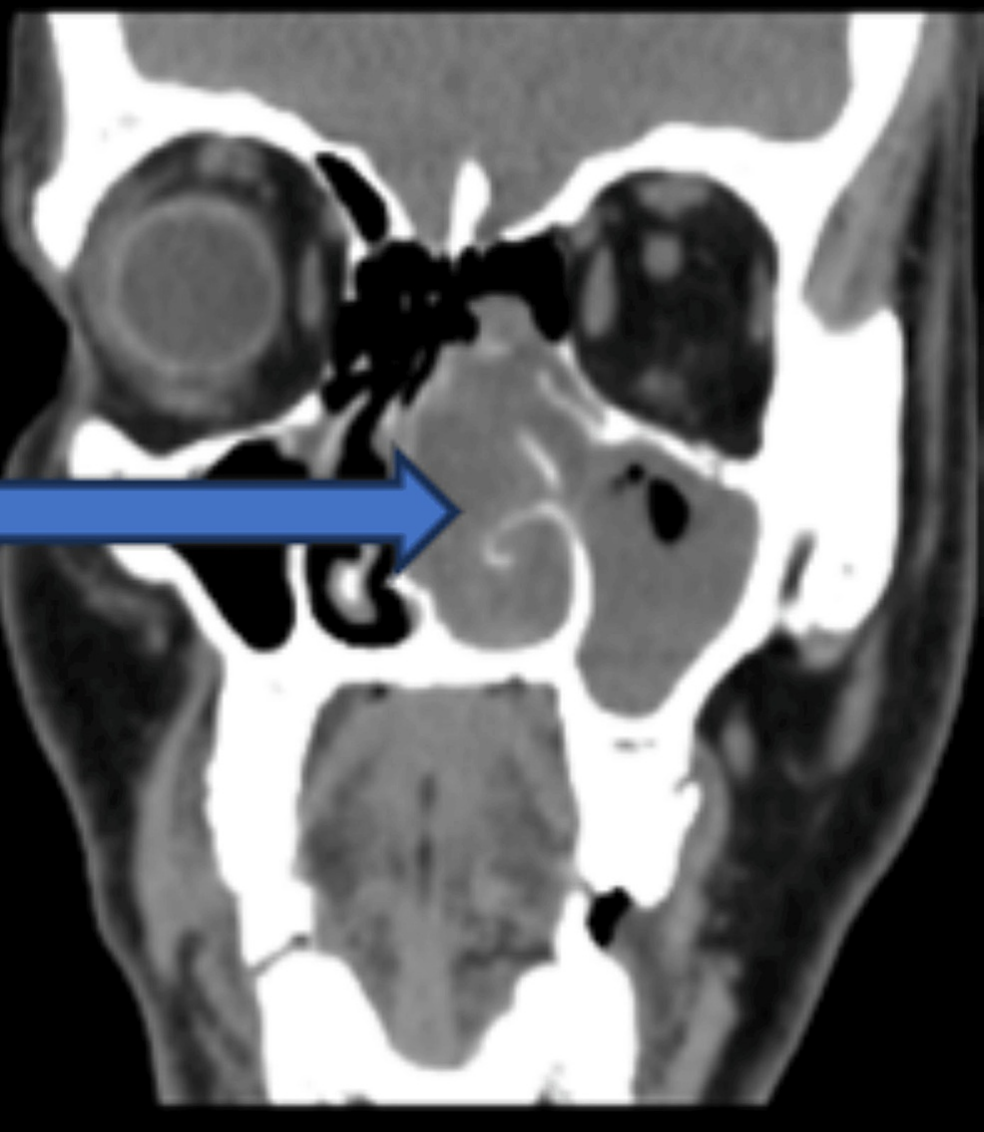
CT scan suggestive of unilateral isodense lesion suggestive of inverted papilloma (solid arrow)

Fungal sinusitis was our study's most common sinonasal pathology because of a sudden spike in mucormycosis after COVID-19. Otherwise, chronic rhinosinusitis and antrochoanal polyps were the most common nasal masses, comprising 14% (n=33) and 12% (n=27), respectively. Nasal polyposis was the predominant sinonasal mass, as per the study by Tondon et al. (64%) and Dasgupta et al. (62.5%) [11,12]. The most common nasal symptoms in our study were nasal obstruction (90%, n=204) followed by nasal discharge (74%, n=169), which is similar to other studies [6,7]. Similar to other studies [7], facial swelling and pain were noticed in 24% (n=54) and 19% (n=44), respectively. Swelling in the oral cavity was present in 10% (n=22) of our cases, which was rarely mentioned in similar literature; this may be due to the maximum number of cases of mucormycosis and advanced angiofibroma in our study. Ocular involvement, like restricted extraocular movement, decreased vision, and proptosis, was seen in 14% (n=31), 14% (n=31), and 7% (n=15), respectively. Other studies also had similar incidences of 10% and 10.7% [6,7], but in some studies, the orbital involvement went up to 21% [11]. In our analysis, the sinonasal undifferentiated tumor was the most common variant (27%, n=6), followed by squamous cell carcinoma (18%, n=4). Most studies conclude that squamous cell carcinoma is the most common variety. Tandon et al. [11] found that squamous cell carcinoma is India's most common histology of sinonasal malignancy, accounting for approximately half of all cases. Another study by Dasgupta et al. [12] also examined sinonasal masses in the Indian population and reported that adenocarcinoma is the third most common mucosal malignancy in the sinonasal region, after squamous cell carcinoma and adenoid cystic carcinoma. This may be due to the small sample size and getting the maximum number of referrals to our center. Almost all our patients had had either a CT scan or an MRI as part of their radiological investigations. For most neoplastic lesions and invasive fungal sinusitis, we prefer MRI over CT because of its ability to differentiate between inflammation and tumor and its greater sensitivity to intracranial extension. However, CT is the selected investigation for chronic rhinosinusitis, inverted papilloma, and other benign diseases [13]. Bony erosion, an extension of the mass into the orbit and beyond the bony confines, can be readily identified through a CT scan (Figure 4). In Figure 5, there is an isodense lesion in the nasal cavity which is suggestive of an inverted papilloma. Other studies also believed that MRI was complementary to a CT scan to evaluate the tumor's soft tissue components and the extent of tumor invasion beyond the bony sinus wall [14-16]. A CT scan is essential for planning surgery and providing a roadmap for endoscopic sinus surgery, and it is also readily available, cheaper, and faster. MRI quickly identifies the invasion and encasement of the cavernous sinus and internal carotid artery [15]. MRI is also helpful in detecting recurrence following surgery.

Limitations of the study

Our study is a retrospective study with a limited sample size, and the duration of the study is shorter. A prospective multicenter study may provide more clear data regarding the clinicopathological variant of sinonasal masses.

## Conclusions

In the present retrospective study, we analyze the various sinonasal masses and their varied clinical presentations. Nasal obstruction followed by nasal discharge were the most common symptoms. The mean age of presentation was 44.5 years. Benign diseases are primarily seen in younger age groups, and malignancy is in the elder group. Sinonasal undifferentiated carcinoma was the most common variant of malignancy in our study, followed by squamous cell carcinoma. CT scans and MRIs were necessary for the proper planning and surgical management of the patients. Histopathology is the gold standard for the appropriate diagnosis of all sinonasal masses.
